# Nonlinear kernels, dominance, and envirotyping data increase the accuracy of genome-based prediction in multi-environment trials

**DOI:** 10.1038/s41437-020-00353-1

**Published:** 2020-08-27

**Authors:** Germano Costa-Neto, Roberto Fritsche-Neto, José Crossa

**Affiliations:** 1 Department of Genetics, “Luiz de Queiroz” Agriculture College, University of São Paulo, São Paulo, Brazil; 2 Biometrics and Statistics Unit, Genetic Resources Program, and Global Wheat Program, International Maize and Wheat Improvement Center (CIMMYT), Mexico, Mexico

**Keywords:** Evolution, Genomics

## Abstract

Modern whole-genome prediction (WGP) frameworks that focus on multi-environment trials (MET) integrate large-scale genomics, phenomics, and envirotyping data. However, the more complex the statistical model, the longer the computational processing times, which do not always result in accuracy gains. We investigated the use of new kernel methods and modeling structures involving genomics and nongenomic sources of variation in two MET maize data sets. Five WGP models were considered, advancing in complexity from a main-effect additive model (A) to more complex structures, including dominance deviations (D), genotype × environment interaction (AE and DE), and the reaction-norm model using environmental covariables (W) and their interaction with A and D (AW + DW). A combination of those models built with three different kernel methods, Gaussian kernel (GK), Deep kernel (DK), and the benchmark genomic best linear-unbiased predictor (GBLUP/GB), was tested under three prediction scenarios: newly developed hybrids (CV1), sparse MET conditions (CV2), and new environments (CV0). GK and DK outperformed GB in prediction accuracy and reduction of computation time (~up to 20%) under all model–kernel scenarios. GK was more efficient in capturing the variation due to A + AE and D + DE effects and translated it into accuracy gains (~up to 85% compared with GB). DK provided more consistent predictions, even for more complex structures such as W + AW + DW. Our results suggest that DK and GK are more efficient in translating model complexity into accuracy, and more suitable for including dominance and reaction-norm effects in a biologically accurate and faster way.

## Introduction

Historically, utilizing the best linear-unbiased prediction (BLUP) has been useful for predicting the performance of unobserved maize hybrids utilizing pedigree or molecular marker relationships of all crosses (Bernardo 1994, 1996). The assessment and prediction of hybrid performance have two main sources of variation: the estimated additive (A) effects among lines based on the variance of the general combining ability of the two parents, and the dominance (D) (and/or epistatic) effects among lines based on the variance of the specific combining ability of the cross between parents (Alves et al. 2019). These two sources are fundamental for prediction based on either pedigree or genome-wide marker information (or both) of the lines forming the single cross. Multi-environment testing (MET) of single crosses facilitates sampling of genotype × environment interactions (GE), as well as additive × environment (AE) and dominance × environment (DE) interactions, and it allows hybrids unobserved in field evaluation to be predicted based on existing data from other observed hybrids derived from related lines.

Prediction-based strategies employing genomic-assisted data (Meuwissen et al. 2001) are responsible for the greatest leaps in genetic gain and reduction of time between selection cycles in both animal- and plant-breeding programs (Crossa et al. 2017; Voss-Fels et al. 2019). Whole-genomic prediction (WGP) focuses on modeling genomic effects due to dense molecular markers related to quantitative-genetics concepts, such as additive and nonadditive variation. WGP studies conducted over the last decade include BLUPs based on different prediction methods, i.e., Ridge Regression and the Genomic Best Linear Unbiased Predictor (GBLUP, VanRaden 2008). These methods have been extensively and intensively employed in maize and wheat hybrid prediction (Windhausen et al. 2012; Lehermeier et al. 2014; Technow et al. 2014; Acosta-Pech et al. 2017; Zhang et al. 2017; Basnet et al. 2019).

However, most genomic hybrid prediction studies ignore GE interaction and do not incorporate environmental covariables to model similarities between environments. In maize, Acosta-Pech et al. (2017) incorporated GE and marker information to predict hybrid performance. A recent study on hybrid wheat investigated the genomic-enabled prediction of single-cross wheat hybrids using models with various combinations of pedigree, markers, and/or their interaction with environments (Basnet et al. 2019). This study on hybrid wheat showed that hybrid prediction accuracy increases when environmental covariables are incorporated, and when additive × environmental covariables and dominance × environmental covariables are included in the GBLUP reaction-norm model (Jarquin et al. 2014). Thus, selection guided by genomic-enabled prediction in multiple environment trials (WGP–MET) can result in optimization of the breeding pipeline by increasing the number of possible hybrids and evaluated environments, especially when aiming to choose the best hybrids for certain environmental conditions, i.e., capable of capturing the effects of the GE. Usually, the WGP–MET models in maize have integrated mainly A and its interaction with the environment (AE). However, more recently, some authors have suggested that the inclusion of dominance effects and their interaction with environments (D and DE) may lead to more accurate WGP-based selection in MET (Wang et al. 2017; Dias et al. 2018; Ferrão et al. 2020).

On the other hand, the use of data derived from environmental typing analysis (e.g., environmental covariable, W) can be an important source to bridge the gap between phenotypic and genomic correlations across MET (Cooper et al. 2014). WGP models including the so-called envirotyping (Xu 2016) analysis can be used to mimic the linear response of the phenotypic performance of genotypes over a certain type of environmental gradient (envirotype), i.e., the reaction norm (Jarquín et al. 2014; Crossa et al. 2017), in which the GE effects are studied as an extension of the GBLUP. The theoretical basis of this modeling approach relies on assuming that the differential envirotype-to-phenotype dynamics for different genotypes drives the GE variation over MET (Millet et al. 2019; Costa-Neto et al. 2020; Porker et al. 2020). In this context, there is a genomic background impacting the phenotypic responses across environments. As the genotypes differ in terms of their allelic constitution, the number of copies of an allele (additivity) and intra-allelic interactions (dominance) are expected to have different degrees of influence on how genotypes respond to environmental variations and how meaningful AW and DW interactions are. For this reason, efforts have focused on a more in-depth search for the genomic causes that are linked to the ecophysiological responses of cultivation, either through genomic association studies (Li et al. 2018) or by genomic prediction considering reaction-norm kernels (Jarquín et al. 2014; Morais Júnior et al. 2018) or whole-genome × envirotyping-based factorial regression models (Ly et al. 2018; Millet et al. 2019).

As already mentioned, the GBLUP (GB) (VanRaden 2008) uses a linear kernel. Other methods consider the complete genetic values of individuals, including both additive and nonadditive (dominance and epistasis) effects, thereby estimating the genetic performance of the lines or hybrids (Crossa et al. 2017). The complexity of applying genomic-based prediction breeding is influenced by various factors acting at different levels. Some of the statistical complexities can be addressed by using semiparametric genomic regression methods to account for nonadditive variation (Gianola et al. 2006, 2011; Gianola and Van Kaam 2008; Morota and Gianola 2014). These methods have been used to predict complex traits with promising practical results (González-Camacho et al. 2012; Pérez-Rodríguez et al. 2012). Semiparametric models often used nonlinear kernel methods for addressing complex gene actions, thus capturing nonlinear relationships between phenotype and genotype. A commonly used kernel is the Gaussian kernel (GK) based on molecular markers (Gianola et al. 2014). Cuevas et al. (2016, 2018) and Souza et al. (2017) showed that using the GK within the multi-environment genomic GE model of Jarquín et al. (2014) led to higher prediction accuracy than the same method with the linear kernel GB. Parametric alternatives for modeling epistasis have also been broadly discussed in the literature (Jiang and Reif 2015; Martini et al. 2016).

Recently, Cuevas et al. (2019) introduced the arc-cosine kernel (AK) function for genome-enabled prediction. The nonlinear AK is defined by a covariance matrix that emulates a deep-learning model with one hidden layer and a large number of neurons. A recursive formula allows altering the covariance matrix stepwise, thus adding more hidden layers to the emulated deep neural network. The AK kernel method has been used in both single-environment and multi-environment models, including genomic × environment interaction (GE) (Crossa et al. 2019; Cuevas et al. 2019). The results of these authors show that AK genomic-enabled prediction accuracy is similar to that of the GK, but AK has the advantage over GK that it is computationally more straightforward, since no bandwidth parameter is required, while performing similarly or slightly better than GK. The tuning parameter “number of layers” required for AK can be determined by a maximum marginal likelihood procedure (Cuevas et al. 2019). Because the AK emulates the action of the deep-learning method, we also name the AK kernel method as Deep kernel (DK) (Crossa et al. 2019). In this paper, we will use AK and DK interchangeably.

Based on the previous studies and on the advantage of using several linear and nonlinear kernel relationships between the covariables (markers and environmental covariables), in this study, we tested the practical aspects of five WGP models. There are only three main-effect models, including environments (E), additive (A), dominance (D), and envirotype (W) (EA, EAD, and EADW), and two are main-effect models plus GE and GW interactions (EAD + GE, EADW + GW) accounting for different genomic and GE and GW covariance structures and using three-kernel methods (GB, GK, and DK). Note that the GE interaction includes EA + ED, whereas GW includes AW + DW. First, we compare the differences between WGP and kernel methods to explain the sources of variation and reduction error variance in MET. Next, we check the computational efficiency of running these models under a Bayesian framework. Finally, we compute the accuracy of each model–kernel method combination using three prediction problems faced by most hybrid maize-breeding programs:

- Predicting hybrids untested in any environment (CV1).
- Predicting hybrids across incomplete trials (the so-called sparse testing, CV2).
- Predicting hybrids in entirely novel environments (CV0).

The three-kernel methods were used on the two types of covariables employed: (1) dense molecular markers, and (2) dense environmental covariables collected in all the environments considered in the two data sets.

## Materials and methods

The “Materials and Methods” are organized as follows. First, in sections “Environmental Typing” and “Maize Data,” we describe the maize data sets used, including genomic and phenotypic data (grain yield, tons per ha), and how environmental data were collected and processed. Next, in sections “Kernel methods” and “Statistical Models” we describe the combinations of the five MET–WGP models, including different structures to accommodate genomic and envirotypic data, and the three-kernel methods used to model them (GB, GK, and DK). Finally, in “Assessing prediction accuracy by cross-validation,” we present the statistical efforts used in testing each combination of the model–kernel method under different experimental network scenarios (CV1, CV2, and CV0).

### Environmental typing

Environmental typing (envirotyping) is a core of procedures used to collect, process, and integrate environmental factors as nongenomic covariates into genetic-informed studies (Cooper et al. 2014; Xu 2016). In this study, a total of 16 environmental factors was used to create what we call envirotype covariable matrix **W** (Table 1).

**Table 1:** List of environmental factors considered in the study, estimated from NASA orbital sensors (Sparks 2018) and processed using concepts from Allen et al. (1998) and Soltani and Sinclair (2012).

Source	Environmental factor	Unit
NASA Power	Top-of-atmosphere insolation	MJ m^−2^ d^−1^
	Average insolation incident on a horizontal surface	MJ m^−2^ d^−1^
	Average downward longwave radiative flux	MJ m^−2^ d^−1^
	Wind speed at 10 m above the surface of the earth	m s^−1^
	Minimum air temperature at 2 m above the surface of the earth	°C d^−1^
	Maximum air temperature at 2 m above the surface of the earth	°C d^−1^
	Dew-point temperature at 2 m above the surface of the earth	°C d^−1^
	Relative air humidity at 2 m above the surface of the earth	%
	Rainfall precipitation (P)	mm d^−1^
Calculated^a^	Effect of temperature on radiation-use efficiency	–
	Evapotranspiration (ETP)	mm d^−1^
	Atmospheric water deficit P-ETP	mm d^−1^
	Deficit of vapor pressure	kPa d^−1^
	Slope of saturation vapor-pressure curve	kPa C° d^−1^
	Temperature range	°C d^−1^
	Global solar radiation based on latitude and Julian Day	MJ m^−2^ d^−1^

^a^Environmental data were collected, processed, and organized by time intervals (phenology) using the functions get_weather(), summaryWTH(), and W.matrix() from the *EnvRtype* package.

First, daily environmental data were obtained from NASA orbital sensors (Sparks 2018). Next, additional variables describing ecophysiological processes (e.g., evapotranspiration, the impact of air temperature on radiation-use efficiency) were computed as extensively described by Allen et al. (1998) and Soltani and Sinclair (2012). Finally, to capture the temporal variation of the environmental information across crop development, the crop cycles were divided into five time intervals:

- From 0 DAE (emergence day) to 14 DAE (appearance of the first leaf, V1).
- From 15 DAE (V1) to 35 DAE (appearance of the fourth leaf, V4).
- From 36 DAE (V4) to 65 DAE (tasseling stage, VT).
- From 66 DAE (VT) to 90 DAE (kernel milk stage, R3).
- From 91 DAE (R3) to 120 DAE (physiological maturity).

These time intervals were defined based on agronomic knowledge of how tropical maize grows in Brazil’s environments. For each variable–phenology combination, we calculated the first (25%), second (50%), and third (75%) percentiles of each combination of environmental variable × time interval across different environments. By using three percentiles, we hope to better capture the statistical distribution of each environmental variable in order to better represent the similarities between environments. In this sense, each combination of environmental variable × time interval × quantile has become an envirotype descriptor of the environmental relatedness. Finally, quality control was done by removing covariables with more than 3 ± SD, where SD is the standard deviation of the covariables across environments (Morais Júnior et al. 2018). This envirotyping pipeline was developed using the core of functions present in the R package *EnvRtype* (available at https://github.com/allogamous/EnvRtype [verified 05 July, 2020]).

### Maize data

The phenotypic data consisted of grain yield (ton/ha) records collected from two data sets of tropical maize hybrids in Brazil (HEL and USP). Both sets include data from Souza et al. (2017) that have been used in previous proof-of-concept studies. Details about the experimental design, cultivation practices, and fundamental statistical analysis are given in Souza et al. (2017) and Alves et al. (2019). Below, we summarize the number of hybrids, the number of environments, and the genomic and envirotyping data used.

### Phenotypes, genotypes, and environmental covariables for the HEL data set

The HEL data set is based on the germplasm developed by the Helix Seeds Company (HEL) in South America. It includes a set of 247 maize hybrids from a core of 452 *F*_1_ hybrids obtained by crossing 106 inbred lines. Those hybrids were evaluated in 2015 in five sites in Brazil (S1–S3 in the southern region and S4–S5 in the mid-west region). Parent lines were genotyped with an Affymetrix Axiom Maize Genotyping Array of 616 K SNPs (single-nucleotide polymorphisms) (Unterseer et al. 2014). Then, standard quality controls (QC) were applied to the data, by removing markers with a call rate ≥0.95. After this process, the remaining missing data in the lines were imputed with the *Synbreed* package (Wimmer et al. 2012) using the algorithms from the *Beagle 4.0* software (Browning and Browning 2008). Finally, markers with a minor allele frequency (MAF) of ≤0.05 were removed, resulting in a total of 52,811 high-quality SNPs. Souza et al. (2017) described both phenotypic and genomic data of inbred lines credited to the Helix Seeds Ltda. Company. According to the geographic coordinates, environmental data were collected for each of the five sites (Supplementary Table S1). At the end of the process described in the “Environmental typing” section, 243 envirotype covariables were obtained (combinations of environmental variables × time intervals × percentiles).

### Phenotypes, genotypes, and environmental covariables for the USP data set

The USP data set is based on the germplasm developed by the Luiz de Queiroz College of Agriculture of the University of São Paulo (USP), Brazil. From 2016 to 2017, a partial diallele experiment involving 49 inbred lines resulting in 906 *F*_1_ hybrids was conducted, and 570 of those hybrids were evaluated across eight environments (E1–E8), involving an arrangement of 2 locations, 2 years, and 2 nitrogen levels. The two sites used in this study involved two distinct biomes with different edaphoclimatic patterns, i.e., Piracicaba (Atlantic Forest, clay soil) and Anhumas (Savannah, silt–sandy soil). At each site, two contrasting nitrogen (N) fertilization levels were used. One experiment was conducted under ideal N conditions and received 100 kg ha^−1^ of N (30 kg ha^−1^ at sowing and 70 kg ha^−1^ in a coverage application at the V8 plant stage), while the second experiment under low N conditions received only 30 kg ha^−1^ of N at sowing. As described in the HEL data set, the parent lines were genotyped with an Affymetrix Axiom Maize Genotyping Array of 616 K SNPs. Markers with a minor allele frequency (MAF) of ≤0.05 were removed. After all QC procedures, a total of 54,113 high-quality SNPs was available for predictions. Environmental data were collected for each of the two sites and 2 years according to the planting date and geographic coordinates (Supplementary Table S1). A nitrogen-management variable was inserted, designating the amount of nitrogen applied in the development cycle (ideal N = 100; low N = 30). At the end of the process described in the “Environmental typing” section, a total of 248 envirotype covariables was obtained.

### Kernel methods

In this study, we tested three methods to estimate the relationship kernels for additive effects (***K***_**A**_), dominance deviations (***K***_**D**_), and envirotype-informed environmental relatedness (***K***_**W**_). The additive effects were modeled from the molecular data, assuming **A** = {0 = *A*^2^*A*^2^; 1 = *A*^1^*A*^2^; 2 = *A*^1^*A*^1^}. Dominance deviations were computed by recoding the matrix of molecular markers for each individual as **D** = {−2*f*_*l*_^2^ = *A*^2^*A*^2^; 2*f*(1 − *f*_*l*_) = *A*^1^*A*^2^; −2*f*(1 − *f*_*l*_)^2^ = *A*^1^*A*^1^} (Vitezica et al. 2013), where *f*_*l*_ is the frequency of the favorable allele at locus *l*. Finally, the envirotyping-based matrix **W** (*q* environments × *k* covariables), with *w* ~ *N*(0,1), was constructed by mean-centering and scaling the environmental information (Environmental typing section). Each of the three-kernel methods is detailed below.

### Benchmark genomic best unbiased predictor

The first method is the traditional GBLUP (GB), where we obtained the covariance matrix from the following expression:

$$GB\!:\,{\boldsymbol{K}} = \frac{{{\boldsymbol{XX}}^\prime }}{{{\mathrm{trace}}\left( {{{\mathbf{XX}}}^\prime } \right)/\mathrm{nrow}\left( {\boldsymbol{X}} \right)}},$$

where ***K*** is a generic representation of the relationship kernel (***K***_**A**_, ***K***_**D**_, and ***K***_**w**_), and ***X*** is a generic representation of the molecular or envirotyping-informed matrix (**A**, **D**, and **W**). By *nrow*(***X***), we denote the number of rows in the ***X*** matrix. The GB method was also used as a benchmark for comparisons with the following two methods.

### Gaussian kernel

The nonlinear Gaussian Kernel (GK) method was the second type of kernel method used in this study. Unlike GB, this kernel is estimated from an exponential relation based on the Euclidean distance ${\boldsymbol{D}}_{ii^\prime }^2 = \mathop {\sum}\nolimits_k {\left( {x_{ik} - x_{i^\prime k}} \right)^2}$ matrix for each pairwise element in ***X*** = {*x*_*i*_, *x*_*i*′_} pondered by its median (a scalar variable, *Q*) and a bandwidth parameter (a scalar variable, *h*) that controls the rate of decay of the covariance between individuals, resulting in

$$GK\!\!:{\boldsymbol{K}} = {\mathrm{exp}}\left( {h{{{\boldsymbol{D}}_{ii^\prime }^2}}/Q} \right),$$

where the diagonal of the GK-based covariance matrix is equal to 1. The bandwidth parameter (*h*) was estimated for each relationship kernel (***K***_**A**_, ***K***_**D**_, and ***K***_**W**_) following the marginal function described in Pérez-Elizalde et al. (2015).

### Deep kernel

The arc-cosine kernel (referred to here as DK) is the third kernel method tested in this study. Cuevas et al. (2019) and Crossa et al. (2019) introduced the use of deep kernels in genomic prediction for multiple environments based on the additive relationship effects. Here we introduce the frequent use of DK for the joint modeling of additive, dominance, and reaction-norm kernels.

The general formulation of the DK method is based on the proposition of Neal (1996) for a Bayesian method for deep artificial neural networks (ANN). After that, Williams (1998) and Cho and Saul (2009) established the relationship between the DK method and a deep neural network with one hidden layer. In this context, the DK method aims to emulate a deep-learning approach, exploring the relationship between individuals within an ***X*** matrix of inputs (e.g., molecular markers, near-infrared data) through the angle (*θ*_*i,i*′_) between two designed vectors of individuals (**x**_*i*_ ⋅ **x**_*i*′_):

$$\theta _{i,i^\prime } = {\cos}^{-1}\left( {\frac{{{\mathbf{x}}_{i} \cdot {\mathbf{x}}_{i^\prime }}}{\Vert{{\mathbf{x}}_i}\Vert\Vert{\mathbf{x}}_{i^\prime }\Vert}} \right),$$

where ⋅ denotes the inner product, and ||**x**_*i*_|| is the norm of hybrid *i*. Cuevas et al. (2019) described a maximum marginal likelihood method used to select the number of hidden layers (*l*) for the DK kernel. As described by Cuevas et al. (2019), the following kernel is positive semidefinite and related to an ANN with a single hidden layer, in which Cho and Saul (2009) describe the activation function as

$$DK^1\left( {{\mathbf{x}}_i,{\mathbf{x}}_{i^\prime }} \right) = \frac{1}{\pi }\Vert {\mathbf{x}}_i\Vert\Vert {\mathbf{x}}_{i^\prime }\Vert J\left( {\theta _{i,i^\prime }} \right),$$

where *π* is the pi constant and *J*(*θ*_*i*_,_*i*′_) is computed by *J*(*θ*_*i*_,_*i*′_) = [sin(*θ*_*i*_,_*i*′_) + (*π* − *θ*_*i*_,_*i*′_)cos(*θ*_*i*_,_*i*′_)]. The DK^1^ is the base kernel defined by a symmetric positive semidefinite matrix, capable of preserving the norm of the entries such as DK(*x*_*i*_, *x*_*i*_) = ||*x*_*i*_||^2^, and *DK*(*x*_*i*_, − *x*_*i*_) = 0 models the nonlinear and orthogonal relationships. Cho and Saul (2009) and Cuevas et al. (2019) present a recursive relationship approach to shape a basic *DK*^1^ into a final DK-emulating ANN hidden layer (*l*), repeating *l* times the interior product

$$DK\!\!:{\boldsymbol{K}} = DK^{\left( {l + 1} \right)}\left( {{\mathbf{x}}_i,{\mathbf{x}}_{i^\prime }} \right) = \frac{1}{\pi }\left[ {DK^{\left( l \right)}\left( {{\mathbf{x}}_i,{\mathbf{x}}_i} \right)DK\left( {x_{i^\prime },x_{i^\prime }} \right)} \right]^{\frac{1}{2}}J\left( {\theta _{i,i^\prime }^{\left( l \right)}} \right),$$

where $\theta _{i,i^\prime }^{\left( l \right)} = {\mathrm{cos}}^{ - 1}\left\{ {DK^{\left( l \right)}\left( {{\mathbf{x}}_i,{\mathbf{x}}_{i^\prime }} \right)\left[ {DK^{\left( l \right)}\left( {{\mathbf{x}}_i,{\mathbf{x}}_i} \right)DK\left( {{\mathbf{x}}_{i^\prime },{\mathbf{x}}_{i^\prime }} \right)} \right]^{ - \frac{1}{2}}} \right\}$. Thus, computing *DK*^(*l*+1)^ at level (layer) *l* + 1 is done from the previous layer *DK*^(*l*)^. To select the number of hidden layer *l* to fill this process for each relationship kernel (***K***_**A**_, ***K***_**D**_, and ***K***_**W**_), at each cross-validation fold, we adopted a maximum likelihood method described by Cuevas et al. (2019).

### Statistical models

The merit of including additive effects (***K***_**A**_), dominance deviation (***K***_**D**_), GE interaction (***K***_**AE**_ and ***K***_**DE**_), and envirotyping-based kinships (***K***_**W**_, ***K***_**AW**_, and ***K***_**DW**_) to estimate reaction norms in MET was assessed using five WGP models. A description of each model structure is given below.

### Model 1: main additive-effect model (EA)

The main additive-effect model (EA) is our benchmark baseline; it is also the simplest modeling structure for WGP in multi-environment trials, following:

1 $${\mathbf{y}}={\mathbf{1}} {\boldsymbol{\mu}} + {\boldsymbol{Z}}_{\mathbf{E}} {\boldsymbol{\beta}}+{\boldsymbol{Z}}_{\mathbf{A}} {\boldsymbol{u}}_{\mathbf{A}} + \boldsymbol{\varepsilon},$$

where ***y*** = [**y**_**1**_,⋯,**y**_**n**_]′ are the vectors of observations collected in each of the *q* environments with *p* hybrids, and **1*μ*** + **Z**_**E**_***β*** is the general mean and the fixed effect of the environments with the incidence matrix ***Z***_**E**_. Genetic variations are modeled by the main additive effects (***u***_**A**_), with ***u***_**A**_ ~ N(**0**,***J***_**q**_⊗***K***_**A**_${{\boldsymbol{\sigma}}^{\mathbf{2}}_{\mathbf{A}}}$), where ***Z***_**A**_ is the incidence matrix for additive effects (absence = 0, presence = 1), ***J***_**q**_ is a *q* × *q* matrix of 1 s, ${{\boldsymbol{\sigma}}^{\mathbf{2}}_{\mathbf{A}}}$ is the variance component for additive effects, and ⊗ denotes the Kronecker Product. Residual deviation (**ε**) was assumed as **ε** ~ *N*(**0**, ***I***_**n**_**σ**^**2**^), where *n* is the number of genotype–environment observations.

### Model 2: main additive plus dominance effects (EAD)

Model EAD (Eq. 2) is a version of model (1) that includes the dominance-deviation effects, as follows:

2 $$\mathbf{y}={\mathbf{1}} {\boldsymbol{\mu}} + {\boldsymbol{Z}}_{\mathbf{E}} {\boldsymbol{\beta}} + {\boldsymbol{Z}}_{\mathbf{A}} {\boldsymbol{u}}_{\mathbf{A}}+{\boldsymbol{Z}}_{\mathbf{D}} {\boldsymbol{u}}_{\mathbf{D}}+ {{\boldsymbol{\varepsilon}}},$$

where ***Z***_**D**_ is the incidence matrix for dominance effects. Note that ***Z***_**A**_ and ***Z***_**D**_ are the same incidence matrix for genotypic effects. However, we included the respective acronyms A and D to facilitate the understanding that we are modeling two different genetic-based sources: additive random variation (as described in 1), and dominance random variation (***u***_**D**_), with ***u***_**D**_ ~ *N*(**0**,***J***_**q**_⊗***K***_**D**_${{\boldsymbol{\sigma}}^{\mathbf{2}}_{\mathbf{D}}}$), where ${{\boldsymbol{\sigma}}^{\mathbf{2}}_{\mathbf{D}}}$ is the variance component for dominance effects.

### Model 3: main-effect EAD plus GE deviation (EAD+GE)

The third model (EAD+GE, Eq. 3) is an update of model (2) accounting for the main effects (***u***_**A**_ and ***u***_**D**_) plus genotype × environment interaction (GE). The inclusion of two multiplicative effects modeled these GE effects, one for additive × environment (AE = ***u***_**AE**_) interaction and the second for dominance × environment (DE = ***u***_**DE**_) interaction:

3 $$\mathbf{y}={\mathbf{1}} {\boldsymbol{\mu}} + {\boldsymbol{Z}}_{\mathbf{E}} {\boldsymbol{\beta}}+{\boldsymbol{Z}}_{\mathbf{A}} {\boldsymbol{u}}_{\mathbf{A}}+{\boldsymbol{Z}}_{\mathbf{D}} {\boldsymbol{u}}_{\mathbf{D}} + {\boldsymbol{u}}_{\mathbf{AE}} + {\boldsymbol{u}}_{\mathbf{DE}}+\boldsymbol{\varepsilon} ,$$

where ${\boldsymbol{u}}_{{\mathbf{AE}}} \sim N\left( {\mathbf0,{\boldsymbol{K}}_{{\mathbf{AE}}}{{\boldsymbol{\sigma }}^{\mathbf{2}}_{{\mathbf{AE}}}}} \right)$ and ${\boldsymbol{u}}_{{\mathbf{DE}}} \sim N\left( {\mathbf0,{\boldsymbol{K}}_{{\mathbf{DE}}}{{\boldsymbol{\sigma }}^{\mathbf{2}}_{{\mathbf{DE}}}}} \right)$, where ${\boldsymbol{K}}_{{\mathbf{AE}}} = {\boldsymbol{Z}}_{\mathbf{E}}{\boldsymbol{I}}_{\mathbf{q}}{\boldsymbol{Z}}_{\mathbf{E}}^\prime \odot {\boldsymbol{Z}}_{\mathbf{A}}{\boldsymbol{K}}_{\mathbf{A}}{\boldsymbol{Z}}_{\mathbf{A}}^\prime$ and ${\boldsymbol{K}}_{{\mathbf{DE}}} = {\boldsymbol{Z}}_{\mathbf{E}}{\boldsymbol{I}}_{\mathbf{q}}{\boldsymbol{Z}}_{\mathbf{E}}^\prime \odot {\boldsymbol{Z}}_{\mathbf{D}}{\boldsymbol{K}}_{\mathbf{D}}{\boldsymbol{Z}}_{\mathbf{D}}^\prime$, and where ${{\mathbf{\sigma }}^{\mathbf{2}}_{{\mathbf{AE}}}}$ and ${{\mathbf{\sigma }}^{\mathbf{2}}_{{\mathbf{DE}}}}$ are the variance components for AE and DE interaction effects, respectively, as suggested by Jarquín et al. (2014), Lopez-Cruz et al. (2015), and Souza et al. (2017); ***I***_**q**_ is an identity matrix denoting a lack of environmental relatedness, and ⊙ denotes the Hadamard product.

### Model 4: main-effect EAD with main envirotype information (EADW)

The next two models are updates of models 2 and 3, including nongenetic information (**W**) from envirotyping data. Jarquín et al. (2014) introduced a strategy to integrate these data in WGP by using environmental covariables to estimate an environmental relatedness kinship (***K***_**W**_) for *q* × *q* environments. Thus, the objective of including the **W** effects is to bridge the gap between the pure genomic information and phenotypic variation observed across the environments. In this context, we tested the incorporation of some envirotype–phenotype relations as the main effects (model 4, Eq. 4) and for GE effects (model 5, Eq. 5 in the next subsection)

4 $${\mathbf{y}} = {\mathbf{1}}{\boldsymbol{\mu}} + {\boldsymbol{Z}}_{\mathbf{E}}{\boldsymbol{\beta}} + {\boldsymbol{Z}}_{\mathbf{A}}{\boldsymbol{u}}_{\mathbf{A}} + {\boldsymbol{Z}}_{\mathbf{D}}{\boldsymbol{u}}_{\mathbf{D}} + {\boldsymbol{u}}_{\mathbf{W}} + \boldsymbol{\varepsilon},$$

where ${{{\boldsymbol{u}}}}_{\mathbf{W}} \sim N\left( {\mathbf{{0}},{{{\boldsymbol{J}}}}_{{{\mathbf{p}}}} \otimes {{{\boldsymbol{K}}}}_{\mathbf{W}}{{\mathbf{\sigma }}^{\mathbf{2}}_{\mathbf{W}}}} \right)$, ${{\mathbf{\sigma }}^{\mathbf{2}}_{\mathbf{W}}}$ is the variance component related to the variation due to envirotype data, and ***J***_**p**_ is a matrix of 1 s with dimension *p* × *p*.

### Model 5: main-effect EADW plus reaction norm for GE (EADW+GW)

The last model (EADW+GW) is an update of (Eq. 3) reaction-norm variation based on the genomic × envirotype effects (GW). In model EADW+GW, we perform the traditional genomic-enabled reaction norm, but discriminating the reaction norm due to additive effects (AW = ***u***_**AW**_) and dominance deviations (DW = ***u***_**DW**_) as follows:

5 $$\mathbf{y} = {\mathbf{1}}{\boldsymbol{\mu}} + {\boldsymbol{Z}}_{\mathbf{E}}{\boldsymbol{\beta}} + {\boldsymbol{Z}}_{\mathbf{G}}{\boldsymbol{u}}_{\mathbf{A}} + {\boldsymbol{Z}}_{\mathbf{G}}{\boldsymbol{u}}_{\mathbf{D}} + {\boldsymbol{u}}_{\mathbf{W}} + {\boldsymbol{u}}_{{\mathbf{AW}}} + {\boldsymbol{u}}_{{\mathbf{DW}}} + \boldsymbol{\varepsilon},$$

where ${\boldsymbol{u}}_{{\mathbf{AW}}} \sim N\left( {\mathbf0,{\boldsymbol{K}}_{{\mathbf{AW}}}{{\boldsymbol{\sigma }}^{\mathbf{2}}_{{\mathbf{AW}}}}} \right)$ and ${\boldsymbol{u}}_{{\mathbf{DW}}} \sim N\left( {\mathbf0,{\boldsymbol{K}}_{{\mathbf{DW}}}{{\boldsymbol{\sigma }}^{\mathbf{2}}_{{\mathbf{DW}}}}} \right)$, with ${\boldsymbol{K}}_{{\mathbf{AW}}} = {\boldsymbol{Z}}_{\mathbf{E}}{\boldsymbol{K}}_{\mathbf{W}}{\boldsymbol{Z}}_{\mathbf{E}}^\prime \odot {\boldsymbol{Z}}_{\mathbf{A}}{\boldsymbol{K}}_{\mathbf{A}}{\boldsymbol{Z}}_{\mathbf{A}}^\prime$ and ${\boldsymbol{K}}_{{\mathbf{DE}}} = {\boldsymbol{Z}}_{\mathbf{E}}{\boldsymbol{K}}_{\mathbf{W}}{\boldsymbol{Z}}_{\mathbf{E}}^\prime \odot {\boldsymbol{Z}}_{\mathbf{D}}{\boldsymbol{K}}_{\mathbf{D}}{\boldsymbol{Z}}_{\mathbf{D}}^\prime$, where ${{\boldsymbol{\sigma }}^{\mathbf{2}}_{{\mathbf{AW}}}}$ and ${{\boldsymbol{\sigma }}^{\mathbf{2}}_{{\mathbf{DW}}}}$ are the variance components for AW and DW interaction effects. Note that in (Eq. 3) we described how to estimate the GE kernels using the Hadamard product between fixed environment and genomic sources. At that point, the GE kernels are estimated using a block diagonal matrix of genomic effects. In contrast, now in (Eq. 5), we replace the identity matrix ***I***_**q**_ with the envirotype-informed kinship ***K***_**W**_, in which a dense matrix models GW kernels. Then it is possible to assume that now there are different relationship levels between genotypes across environments according to the envirotyping-based kinships.

### Assessing prediction accuracy by cross-validation

In this study, three cross-validation schemes were used to evaluate the predictive ability (PA) of each model–kernel method combination. The first scheme aimed to quantify the accuracy of WGP models when predicting new genotypes within the experimental network, i.e., maize hybrids not yet tested in any environment. This validation scheme is called CV1, which was run 50 times using random samplings of 70% of phenotypic information, while the remaining data were predicted. The second scheme aimed to quantify the predictability of WGP models under sparse experimental network conditions. In contrast to CV1, in this scheme (CV2), the sparse phenotypic information of one genotype not evaluated in one environment, but evaluated across other different environments, can help increase PA. For this scheme, 50 random repetitions were also used, but sampling 70% of the phenotypic information (genotype–environment combinations) as the training population, and the remaining 30% as the test population. Finally, the third scheme aimed to quantify WGP models’ ability to predict new environmental conditions. For this, we adopted a leave-one-environment-out scheme (CV0).

PAs were evaluated at two levels: (1) the model level, in which we computed Pearson’s correlation between observed (*y*) and predicted values ($\widehat y$) and, finally, for CV0, the general average of these correlations, and (2) the genotype level, in which we computed the predictability related to the observed and predicted performance of a genotype in all environments. The standard error (*SE*) was computed for each average PA following ${SD} = {SD} \times \sqrt {\frac{1}{{n}} + \frac{{{n}_2}}{{{n}_1}}}$, where *SD* is the standard deviation of the correlations, *n* = *pq* for *p* genotypes (hybrids) and *q* environments, and *n*_1_ and *n*_2_ denote the size of the training and testing populations for each CV scheme (Bouckaert and Frank 2004).

### Hierarchical Bayesian modeling

Genomic predictions were performed using the Bayesian Genotype plus Genotype × Environment (*BGGE*) package (Granato et al. 2018). This package contains a function called “BGGE()” that solves mixed linear models through hierarchical Bayesian modeling. Below, we briefly describe the main distributions and priors used by this package. First, each variance–covariance matrix (***K***) is reparametrized using an eigen-decomposition procedure suggested by De Los Campos et al. (2010), ***K*** = ***USU*′** where ***S*** is a diagonal matrix with n nonzero eigenvalues and ***U*** is an orthogonal matrix with eigenvectors; hence, an orthogonal transformation suggested by Cuevas et al. (2014). In this transformation, the phenotypic parametrization is represented as ***d*** = ***U*′*y***, and any kernel-based random effect (***b*** = ***U*′*u***) and error variation (***e*** = ***U*′ε**) is now represented into a reparameterized normal distribution as ${\boldsymbol{b}} \sim N\left( {0,{\boldsymbol{U}}^\prime {\boldsymbol{KU}}{{\boldsymbol{\sigma }}^{\mathbf{2}}_{{{\boldsymbol{u}}}}}} \right) = N\left( {0,{\boldsymbol{S}}{{\boldsymbol{\sigma }}^{\mathbf{2}}_{{{\boldsymbol{u}}}}}} \right)$ and ${\mathbf{e}} \sim N\left( {0,{\boldsymbol{U}}^\prime {\boldsymbol{U}}_\varepsilon ^2} \right) = N\left( {0,{\boldsymbol{I}}{\boldsymbol{\sigma}} _\varepsilon ^2} \right)$. Both processes are employed to increase the computational efficiency of the subsequent steps. Thus, the distribution of the transformed data is now given by

$$f\left( {\left. {\boldsymbol{d}} \right|{\boldsymbol{b}},{\boldsymbol{\sigma}} _\varepsilon ^2} \right) = \mathop {\prod}\limits_{i = 1}^n {N\left( {\left. {d_i} \right|b_i,\sigma _\varepsilon ^2} \right)},$$

where the acronym *i* now denotes each random effect (variance–covariance) considered (e.g., for additive, dominance, and envirotyping data). As this Bayesian linear model assumes $p\left( {\left. {\boldsymbol{u}} \right|{{\sigma}} _u^2} \right) = N\left( {\left. {\boldsymbol{u}} \right|0,{\boldsymbol{K}}{\boldsymbol{\sigma }}_u^2} \right)$, the conditional of any *b*_*i*_ is given as $p\left( {\left. {b_i} \right|\sigma _u^2} \right) = N\left( {\left. {b_i} \right|0,\sigma _u^2s_i} \right)$, where *s*_*i*_ are the eigenvalues. Thus, the BGGE package assumes that conjugate prior distribution of $\sigma _u^2$ and $\sigma _\varepsilon ^2$ is given by inverse chi-squared with ${\mathrm{p}}\left( {\sigma _u^2} \right) \sim \chi ^{ - 2}\left( {\nu _u,Sc_u} \right)$ and ${\mathrm{p}}\left( {\sigma _\varepsilon ^2} \right) \sim \chi ^{ - 2}\left( {\nu _\varepsilon ,Sc_\varepsilon } \right)$, respectively, in which *v*_*u*_ and *v*_*ε*_ denote the degree of freedom, and *Sc*_*u*_ and *Sc*_*ε*_ the scale factors for ***u*** and ***e***. Then, the joint posterior distribution $\left( {{\boldsymbol{J}} = {\boldsymbol{b}},\sigma _u^2,\,\sigma _\varepsilon ^2} \right)$, given the parameters (***P*** = ***d***, *v*_*u*_, *v*_*ε*_, *Sc*_*u*_, *Sc*_*ε*_ and ***S***), is

$$p({\boldsymbol{J}}\mid {\boldsymbol{P}}) \propto\left\{\prod_{i=1}^{n} N\left(d_{i} \mid b_{i}, \sigma_{\varepsilon}^{2}\right) N\left(b_{i} \mid 0, \sigma_{u}^{2} s_{i}\right)\right\} \times\, \chi^{-2}\left(\sigma_{u}^{2} \mid v_{u}, v_{u} S c_{u}\right) \times \chi^{-2}\left(\sigma_{\varepsilon}^{2} \mid v_{\varepsilon}, v_{\varepsilon} S c_{\varepsilon}\right)$$

Finally, BGGE uses the Markov chain Monte Carlo (MCMC) procedure to generate the conditional distributions through a Gibbs sampler. Details of this package and functions are given in Granato et al. (2018). For all combinations of model and kernel methods tested in this study, the MCMC through a Gibbs sampler was performed for 10,000 iterations with the first 1000 cycles removed as burn-in with thinning equal to 2.

## Results

### Differences in explaining the sources of variation

**Fig. 1:**
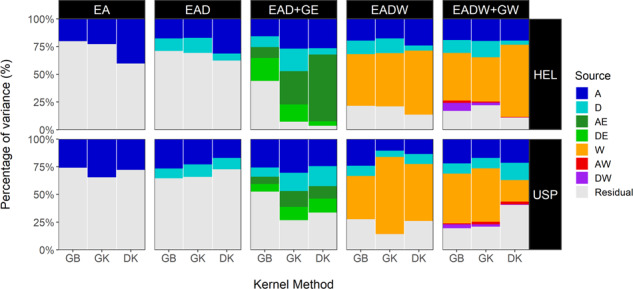
Partition of the variance components related to the different genetics, environmental, and residual sources of variation. Each panel is based on the combination of the five WGP models (vertical titles) built with the three different kernel methods (*x* axis) over HEL and USP data sets (horizontal titles).

When including new sources of variation, as well as when modeling these sources by different kernels, it is expected that differences in the proportion of variance explained by WGP can be detected (Fig. 1 and Supplementary Tables S2 and S3).

Additive effects (A) are the main source of genomic variation in all models. In the EA model, the A effects are best explained by the DK (HEL set) and GK (USP set) kernels. The inclusion of D effects increased the genomic prediction ability to explain phenotypic variation. In the additive-dominant model (EAD), the use of GK was more efficient in capturing dominance effects (D, light-blue color in Fig. 1) in both data sets. For the HEL set, using the DK kernel to model dominance effects resulted in an increase in the additive genomic variance and a reduction in the residual variance. In the USP set, the dominance effects were better modeled by GK, while the traditional GB kernel captured the total genomic effects (A + D) better.

The biggest differences between kernel methods were observed in the most complex models involving GE interaction and envirotyping data. In GB, it is possible to verify that the interaction between home and environment (DE, light-green color in Fig. 1) was an important variation to describe the phenotypic variance in the tests. In general terms, in models with GE interaction (GE = AE + DE), the GK kernel was more efficient in explaining the main additive and dominant effects in both sets. However, for the HEL set, the DK kernel was more efficient in reducing the residual variance by capturing the effects of additive × environment interaction (green color in Fig. 1) better. Upon comparing GB with GK and DK, these last two kernels increased the variance explained by the genomic prediction model.

Reaction-norm models tend to capture a large amount of variance and drastically reduce the residual error. The inclusion of the main effect of envirotyping-informed relationships (W, orange color in Fig. 1) produced similar results as those observed for models with EAD + GE effects. There was a drastic reduction in the residual variation of EADW benchmarked with the EAD model for all models and kernels. In models involving the reaction norm for the effects of GW = AW + DW (model 4, EADW + GW) for the HEL set, there was an increase in the capacity of the models to explain D effects using GB and GK, especially in the reaction norm for dominance (DW, purple colors in Fig. 1) using GB. When a reaction norm for AW + DW is integrated, most of the phenotypic variance is explained by nongenomic effects from W. For the USP data set, the DK kernel was more conservative in modeling W effects; in contrast, it was better able to model the main A, D, and AW interaction. Despite this, it was the model whose proportion of residual variance was the highest.

### Computational efficiency

The processing time of the models is a key issue for their widespread use in WGP–MET. All the benefits of complex models involve different genomic and environmental structures, but are computationally costly and unlikely to achieve wide approval by plant breeders. Here we calculated the processing time of a Bayesian Markov chain involving 10,000 iterations for each model and kernel method combination involving all the phenotypic data of *p* hybrids in *q* environments in both data sets (Table 2).

**Table 2:** Total time (in seconds) to execute a Markov chain containing 10,000 iterations using the BGGE package for each combination of genomic prediction model, kernel method, and maize data set.

Set	Model	GB	GK	DK
HEL	EA	47	58 (+19%)	54 (+13%)
	EAD	97	101 (+4%)	88 (−10%)
	EAD+GE	134	139 (+4%)	126 (−6%)
	EADW	175	139 (−26%)	126 (−39%)
	EADW+GW	330	294 (−12%)	280 (−18%)
USP	EA	660	718 (+8%)	655 (−1%)
	EAD	1442	1341 (−8%)	1360 (−6%)
	EAD+GE	1684	1585 (−6%)	1600 (−5%)
	EADW	2440	1884 (−30%)	2202 (−11%)
	EADW+GW	4368	3800 (−15%)	4087 (−7%)

Values in parentheses denote the relative gain/reduction in computational time using GK and DK in comparison with the same model based on GB.

As expected, more complex models tend to take more processing time, which can range from 47 s (EA) to 330 s (EADW + GW) in smaller data sets like HEL (*q* = 5, *p* = 247), or 660 s (EA) and up to 4368 s (EADW + GW) in larger data sets, such as USP (*q* = 8, *p* = 570). In the simplest model (EA), GB is faster than GK and DK in both sets. However, as the complexity of the models increases, GB becomes increasingly slower than DK and GK. The DK kernel is significantly faster than GB and GK, even running the same Markov chain in 39% less time than GB. It is possible to run more complex models using GK and DK in similar time as simpler models using GB. For the USP set, it was possible to see that GK was faster than DK under most scenarios, even running a more complex model with environmental data and additive-dominant effects (EADW) at almost the same speed as a traditional GE interaction model via GB.

### Accuracy in the HEL set

Table 3 presents the results from the three cross-validation schemes (CV1, CV2, and CV0) for each model–kernel method combination in the HEL set. For CV1 and CV2, the simplest model structures (EA and EAD) were unable to produce an accurate prediction of grain yield concerning the most complex models (EAD+GE, EADW, and EADW+GW). The inclusion of D effects (EAD) led to an increase in PA for CV1 schemes. In contrast, there was a reduction in PA for CV2 when the main D effects were included (EAD model). For the EA and EAD models, there were no great differences in PA between the three-kernel method adopted in both CV1 and CV2 schemes.

**Table 3:** Average correlations between predicted and observed values for grain yield (tons per ha) using five statistical models, three kernel methods, and cross-validation schemes (CV1, CV2, and CV0) for a HEL maize set with 247 hybrids in five environments.

CV	Kernel	Model				
		EA	EAD	EAD+GE	EADW	EADW+GW
**CV1**	**GB**	0.247	0.345	0.220	**0.832**	0.819
		(0.028)	(0.023)	(0.037)	(0.007)	(0.006)
		–	28%	−12%	70%	70%
	**GK**	0.306	0.350	**0.871**	0.831	0.824
		(0.024)	(0.021)	(0.006)	(0.016)	(0.007)
		–	13%	65%	63%	63%
	**DK**	0.305	0.338	0.669	**0.822**	**0.819**
		(0.016)	(0.020)	(0.019)	(0.008)	(0.007)
		–	10%	54%	63%	63%
**CV2**	**GB**	0.231	0.208	0.132	**0.839**	0.824
		(0.033)	(0.026)	(0.041)	(0.007)	(0.007)
		–	−11%	−75%	73%	72%
	**GK**	0.240	0.197	**0.892**	0.838	0.835
		(0.029)	(0.025)	(0.008)	(0.017)	(0.007)
		–	−22%	73%	71%	71%
	**DK**	0.209	0.172	0.734	**0.839**	**0.836**
		(0.022)	(0.031)	(0.006)	(0.009)	(0.009)
		–	−21%	72%	75%	75%
**CV0**	**GB**	0.402	0.558	0.551	**0.567**	0.537
		(0.059)	(0.045)	(0.046)	(0.041)	(0.046)
		–	28%	27%	29%	25%
	**GK**	0.505	0.560	**0.569**	**0.568**	**0.567**
		(0.055)	(0.047)	(0.034)	(0.041)	(0.042)
		–	10%	11%	11%	11%
	**DK**	0.533	**0.570**	**0.571**	**0.572**	**0.569**
		(0.064)	(0.049)	(0.036)	(0.042)	(0.041)
		–	7%	7%	7%	6%

Standard error values (*SE*) and the predictability gains in relation to the baseline model (EA) are given parentheses and %, respectively. Bold numbers denote the best models for each kernel method.

For the most complex models, however, there was a drastic difference between kernel methods. For model 3 (EAD + GE), the GB was unable to reproduce the GE effects of AE and DE interactions. On the other hand, the GK and DK kernels satisfactorily exploited the AE + DE effects, translating model complexity in PA, with increments ranging from 54% (DK at CV1) to 73% (GK at CV2) compared with the baseline EA model. EAD+GE outperformed the best GB-based models for both CV1 and CV2 schemes (EADW, with *r* = 0.832 for CV1 and *r* = 0.839 for CV2) based on GK (*r* = 0.871 in CV1 and *r* = 0.892 in CV2). The reaction-norm models (EADW and EADW + GW) using DK were similar to the GB models for both CV1 and CV2, but it took less computational time to run them (see Table 2).

The results for CV0 are presented in the last part of Table 3. As expected, the PA values were higher than CV1 and CV2 because this scheme uses much more phenotypic information than the other schemes. However, in CV0, it faced the problem of predicting the performance of the hybrids in an entirely new environment. All GK- and DK-based models outperformed the GB models. The use of complex structures from environmental data was useful for GB kernels, but in contrast, modeling structures based on GK and DK led to a similar result just by the inclusion of dominance effects (EAD for DK) and GE interaction (EAD for GK and DK). In summary, it was possible to achieve the same results for reaction-norm GB using dominance effects or GE interaction in DK.

### Accuracy in the USP set

Table 4 shows the results from the three cross-validation schemes (CV1, CV2, and CV0) for each model–kernel method combination in the USP set. As expected, the PA values were higher for CV0, followed by CV2 and CV1. In this last scheme, the inclusion of D effects led to an increment in PA for all kernels, except GK. As observed in the HEL set, model 3 (EAD+GE) based on GB was not satisfactory in exploring GE interaction. PA values were higher in models including nongenetic effects derived from envirotyping data (EADW and EADW+GW) than in pure genomic models (EA, EAD, and EAD+GE). In CV1, the best GB model (EADW+GW) was the same as the EAD+GE model using GK and DK. This last kernel led to greater PA values when some envirotyping data were used (*r* = 0.822 for EADW and *r* = 0.818 for EADW+GW).

**Table 4:** Average correlations between predicted and observed values for grain yield (tons per ha) using five statistical models, three kernel methods, and cross-validation schemes (CV1, CV2, and CV0) for a USP maize set with 570 hybrids in eight environments.

CV	Kernel	Model				
		EA	EAD	EAD + GE	EADW	EADW + GW
**CV1**	**GB**	0.306	0.328	0.287	**0.658**	**0.669**
		(0.019)	(0.018)	(0.020)	(0.010)	(0.009)
		–	7%	−7%	53%	54%
	**GK**	0.324	0.323	**0.673**	**0.671**	**0.689**
		(0.018)	(0.017)	(0.009)	(0.010)	(0.009)
		–	0%	52%	52%	53%
	**DK**	0.305	0.338	0.669	**0.822**	**0.819**
		(0.016)	(0.02)	(0.009)	(0.008)	(0.007)
		–	10%	54%	63%	63%
**CV2**	**GB**	0.339	0.367	0.316	0.714	**0.731**
		(0.015)	(0.012)	(0.016)	(0.005)	(0.006)
		–	8%	−7%	53%	54%
	**GK**	0.370	0.362	0.733	0.730	**0.751**
		(0.013)	(0.012)	(0.005)	(0.005)	(0.005)
		–	−2%	50%	49%	51%
	**DK**	0.349	0.349	**0.891**	0.724	0.745
		(0.014)	(0.014)	(0.008)	(0.007)	(0.006)
		–	0%	61%	52%	53%
**CV0**	**GB**	0.335	0.425	0.427	**0.489**	**0.515**
		(0.014)	(0.015)	(0.016)	(0.088)	(0.105)
		–	21%	22%	32%	35%
	**GK**	0.406	0.429	**0.456**	**0.498**	**0.493**
		(0.015)	(0.015)	(0.021)	(0.098)	(0.090)
		–	5%	11%	19%	18%
	**DK**	0.403	0.428	**0.458**	**0.526**	**0.566**
		(0.015)	(0.016)	(0.034)	(0.098)	(0.092)
		–	6%	12%	23%	29%

Standard error values (*SE*) and the predictability gains in relation to the baseline model (EA) are given in parentheses and %, respectively. Bold numbers denote the best models for each kernel method.

The DK method was also efficient in exploring the main D effects (*r* = 0.338 in EAD) and GE interaction (*r* = 0.669 in EAD+GE, an increment of 54% compared with the EA model). However, in the CV2 scheme, it was possible to see how the DK method was efficient in providing a more computationally efficient approach that captures AE + DE effects better. Model EAD+GE based on DK achieved the highest PA value for all CV schemes (*r* = 0.891), while the best GB model (EADW+GW) had a PA value equal to *r* = 0.731. GK was also efficient in exploring genomic AE+DE effects (*r* = 0.733) and the inclusion of nongenomic reaction-norm effects (*r* = 0.751). Finally, in CV0, it was possible to measure the models’ ability to predict novel environments. The DK outperformed the GK and GB kernels and produced more precise predictions incorporating D, GE effects, and envirotyping data.

### Resolution of genomic prediction for specific hybrids

Most studies involving WGP–MET only assess the accuracy of the models in predicting the entire data set over a specific cross-validation scenario, as presented in the previous sections. Here we introduce the concept of resolution of the WGP models by evaluating the models’ ability to reproduce the phenotypic performance of specific maize hybrids within MET. The phenotypic data used as a training set in these models were obtained from (*q* − 1) environments, where the one-environment-out is a novel growing condition in which the hybrid was not tested (CV0). Thus, the following results are a scenario in which maize breeders have already evaluated the genotypes across MET, but are interested in making predictions of the phenotypic performance of desirable target hybrids.

**Fig. 2:**
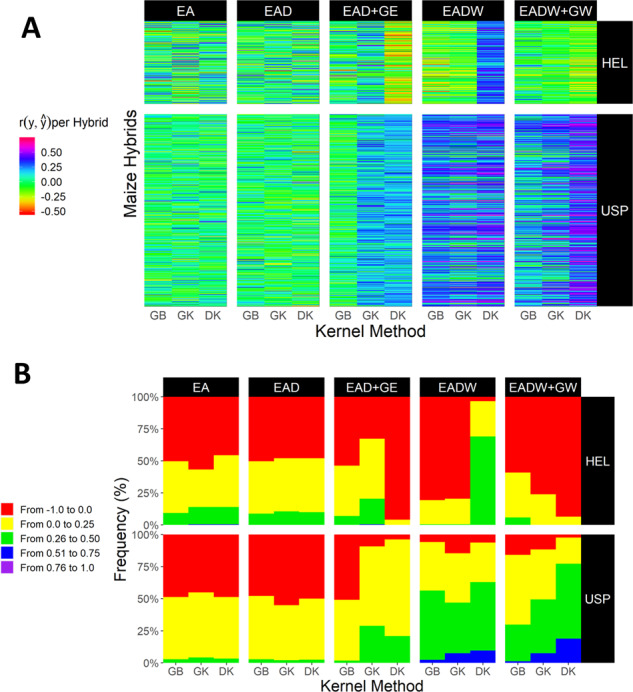
Resolution of the genomic-enabled models and kernel methods in predicting genotypes in novel environments. **a** Predictive ability of specific hybrids (each row) involving (*q* − 1) tested environments plus a one novel environment for sets HEL and USP, respectively; **b** typology of predictive abilities for sets HEL and USP, respectively. Predictive ability values are represented from warm colors (red, worst results) to cold colors (blue and purple, better results).

Figure 2 presents the PA values for specific hybrids (rows) (Fig. 2a) and the typology (distribution pattern) of those predictions for each model–kernel method combination (Fig. 2b) and each data set (HEL and USP). For both data sets, it is possible to observe that different model–kernel method combinations can predict different hybrids (Fig. 2a). The same hybrid can be well predicted by a simpler model, but not predicted by a more complex model. In contrast, the inclusion of more complex structures, such as the reaction norm, may not always lead to a better description of a target hybrid. For this reason, we analyzed the typology of those predictions (Fig. 2b), aiming to observe which model–kernel method combinations are more accurate in reproducing most of the hybrids.

The simplest modeling structures (EA and EAD) are incapable of reproducing the performance of almost 50% of the hybrids in both sets (green colors in Fig. 2a and red colors in Fig. 2b). For those models, the use of any kernel method has led to almost the same result. The greatest differences are observed when genotype × environment (GE) interaction effects are included (EAD+GE). GB was the worst kernel method for exploring the GE effects and translating them into a higher resolution of WGP. GK was the best kernel method, as shown in the blue color of Fig. 2a and yellow bars in Fig. 2b. DK was very efficient in the USP set, but it was not observed in the HEL set. An explanation of that may be that the DK was overfitted for the HEL set, with a smaller sample of phenotypic data.

The higher resolution of WGP was achieved by the inclusion of envirotyping-based data to model the main environmental effects (EADW) or reaction-norm variation (EADW + GW) into the additive-dominance models. For the HEL set, the EADW model with DK was the best modeling approach, with the highest PA values (blue and dark-blue colors in Fig. 2a) and with less than 4% of the hybrids not well predicted (values above 0, red bars in Fig. 2b). The most frequent PA type had values from 0.26 to 0.50 (green colors in Fig. 2b). For the USP set, all kernel methods drastically improved the resolution of WGP for both EADW and EADW + GW models (Fig. 2a). The model–kernel method differences were better represented in the EADW and EADW + GW panels in Fig. 2b. GK outperformed GB in increasing the frequency of higher PA values (green and blue bars in Fig. 2b). In the same way, DK outperformed GK for both EADW and EADW+GW models. The typology of the EADW+GW model based on DK presents negative PA values at a frequency of less than 3%. Conversely, the predominant type is between 0.26 and 0.50 (~50% of the hybrids) and values between 0.51 and 0.75 (~20% of the hybrids).

### Accuracy trends for novel environments

**Fig. 3:**
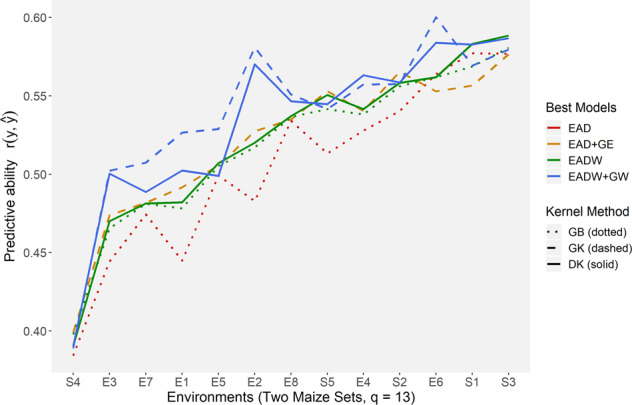
Joint accuracy trends of best combinations of kernel method and model in predicting novel environments (CV0) for both maize data sets (HEL and USP). On the *X* axis, the environments were ordered from less predictable (S4) to higher predictable (S3). Environments with the acronym S denote sites (from 1 to 5, in the HEL set) and with E denoting environments (from 1 to 8, in the USP set).

Based on the results presented in the previous section, we selected six model–kernel method combinations to be jointly evaluated in terms of their capacity to predict novel environments (Fig. 3). It was difficult to determine which models were better in the less predictable environment (S4, from the HEL set). However, as the predictability of environments increases, it is possible to better understand how different kernel methods and models can reproduce the phenotypic information of a novel environmental condition. The use of the main-effect additive-dominant GB (GB-EAD, red dotted line in Fig. 3) was the most unstable framework in CV0. In contrast, the incorporation of envirotypic data (GB-EADW, green dotted line in Fig. 3) was responsible for increasing the PA for less predictable environments and stabilizing the response of the additive-dominant model in reproducing novel environments.

The GB-EADW model had a similar performance as models DK-EADW (solid green line in Fig. 3) and GK-EAD+GE (golden dashed line in Fig. 3). In contrast to the other models, the inclusion of the AW and DW effects (blue lines) combined with the GK (dashed blue line) and DK (solid blue line) kernels increased the PA for all environments, especially for E2, E3, and E6, corresponding to ideal N conditions in Piracicaba in 2016, low N conditions in Anhumas in 2016, and ideal N conditions in Piracicaba in 2017. Between these two reaction-norm models, the GK outperformed the DK and achieved higher PA values for most of the environments.

## Discussion

In this study, we presented the first report on (1) the joint modeling of additive and dominance effects with reaction-norm variation, (2) the modeling of these effects performed by Gaussian Kernel and Deep Kernel, and (3) their comparison with benchmark GBLUP-based modeling. We reported that the Gaussian Kernel and Deep Kernel outperformed GBLUP in reducing the computational time, and increased the PA for all testing scenarios in tropical maize. Below, we discuss how the use of dominance effects and envirotyping-aided reaction-norm modeling is the main bottleneck for increasing PA in GBLUP-based models over MET. In addition, we suggest that the Gaussian Kernel is the best alternative to model dominance variation and translate it into PA gains. Finally, we discuss that Deep Kernels also have greater potential to be used on large-scale genomics and “enviromics” (the core of envirotyping-based big data). They are faster, capture better additive and dominance effects, and have greater predictive accuracy than other kernels under several prediction conditions faced by maize breeders in the development of hybrids.

### Importance of dominance effects in GBLUP

In all the predicted scenarios evaluated (CV1, CV2, and CV0), the models integrating both genomic and envirotyping data tended to have better ability to reproduce the phenotypic performance of maize hybrids. As reported in other studies in plants, the inclusion of dominance effects in traditional WGP–MET resulted in increased predictive accuracy in models based on GBLUP compared with other methods. Azevedo et al. (2015) showed that GBLUP-based models outperform methods such as Ridge Regression (e.g., BayesA, Bayes/LASSO) in modeling A+D genetic effects in simulated populations. Dias et al. (2018) demonstrated that GBLUP models containing A+D effects doubled the predictive capacity for grain yield in maize under diverse environmental conditions, such as environments with limited water availability (i.e., drought-stress screening trials). In a study based on simulations for a pine-breeding population, De Almeida Filho et al. (2016) suggest that the gains in predictive capacity obtained by the A+D model compared with the model based only on A are only relevant if the D effects explain at least 20% of the phenotypic variation. Here we show that not only the main D effects but also their interaction with the environment (D+DE and D+DW) was responsible for 25–40% of the phenotypic variation in both maize sets. This can explain the excellent results found in this study, especially when the GK and DK kernels, better able to capture such effects, are used in the prediction. Despite the aforementioned factors, the inclusion of D effects is essential for the accurate modeling of phenotypic variation in species with some degree of heterosis (Technow et al. 2014), such as in this study using F_1_ single crosses.

For the prediction of new environments (CV0) in our study, we observed a leap in accuracy from 0.402 to 0.558 (+39%) in HEL, and from 0.335 to 0.425 (+27%) in USP, which can be explained by the fact that dominance effects are important for controlling the stability and adaptability of single-maize hybrids, making them more predictable. However, without any envirotyping data, the possible accuracy achieved by those models for grain yield is limited. This trait is quantitatively inherited, controlled by many genes of small effects, and has strong epistatic relationships with several other traits highly influenced by the environment, such as the number of grains per ear and ear size. In this sense, within MET, the use of dominance effects produced by a covariance-based kinship may not be enough. Details about how dominance effects were better modeled using Gaussian kernel and Deep kernel are discussed in the next few sections.

### Envirotyping data are a limit breaker for MET GBLUP

For the prediction of novel maize hybrids, the greatest leap in accuracy in GBLUP was due to the ability to integrate the envirotyping information in the modeling of the reaction norm at the level of additive effects (AW) and dominance deviations (DW). This fact suggests that dominance effects are indispensable for a deep understanding of the genomic causes driving genomic × environment (GE) interaction for each hybrid. In the HEL data set, the models including only the main effects (EADW) had a performance similar to that of the models containing GW effects (EADW+GW). This can be explained by the fact that, in this data set, GE interaction was not as important as in USP; therefore, the inclusion of envirotyping data was enough to adjust the genomic responses according to the degree of similarity between environments.

In contrast to the reaction-norm models (EADW and EADW+GW), the GBLUP was not efficient in reproducing GE interactions in the models assuming that environments are not related (EAD and EAD+GE). Thus, the inclusion of envirotyping data (W and GW) may be the only alternative to breaking the limits of PA achieved in MET–WGP employing the benchmark GBLUP kernel in maize. The prediction of novel environments is restricted to models including envirotyping data, even if the dominance effects are taken into account. However, despite the higher accuracy gains achieved by including W or GW effects, those models are computationally expensive and were outperformed by other kernel methods employing the same molecular and envirotyping data.

### DK and GK better model interaction effects

In contrast to GBLUP, both Gaussian kernel and Deep kernel methods were successful in reproducing genomic × environment (GE) interaction, even in those models that assume that environments are not related. In the case of the Gaussian kernel, its higher efficiency in capturing interaction effects from intra-allelic (dominance) and whole GE interaction may be because such effects are better understood in terms of nonlinear relationships and Euclidean distances, and not as linear covariances as given in GBLUP. The use of covariances to estimate an existing relationship between individuals has its origins in the work of VanRaden (2008), which focused on modeling pedigree and additive–genomic effects. On the other hand, the Gaussian kernel assumes a diagonal equal to 1.0 and an off-diagonal based on the Euclidean distance regulated by a bandwidth factor. Thus, the genetic sense of this matrix property for an F_1_ hybrid individual is that the effects of dominance are the highest within an individual. The relationship between individuals depends on the distance between the effects of intra-allelic interaction shared between related individuals. Similarly, the GE interaction corresponds to whole-genomic effects being differentially activated/deactivated, for each genotype, as a function of the total existing environmental inputs (E → GE). The inclusion of envirotyping data leads to a deeper understanding of this dynamic, which is converted as a function of the known environmental inputs, and of how a particular genomic response of different genotypes is distanced. On the other hand, the use of a Deep kernel seeks to model the genomic relationship matrix based on emulating hidden layers capable of capturing different levels of depth of the same genomic effect. In this work, we introduced simultaneous and independent modeling of hidden layers for additive and dominance effects, which capture different relationship patterns between individuals based on the phenotypic information provided in the training set. Unlike the Gaussian kernel, the diagonal elements of the Deep kernel are not identical (Supplementary Fig. S1–S3), for they express heterogeneous variances of the genetic and environmental effects. This may be why the Gaussian kernel overcame the Deep kernel in the EAD + GE models in CV1 and CV0. As for CV2, the Deep kernel benefited from the fact that the borrowing of phenotypic information across multiple environments helped shape the covariance structure carried out by the hidden layers.

### Approaching envirotype-to-phenotype modeling

In this work, we also introduce the use of the nonlinear methods (Gaussian kernel and Deep kernel) in the modeling of genomic and nongenomic (environmental) kinships. Since the first report of a genomic-enabled prediction considering the reaction norm, as proposed by Jarquín et al. (2014), the environmental relationship kernel (***K***_**W**_) was modeled by the benchmark GBLUP approach. Here we show that the similarity among environments is better modeled in terms of Gaussian processes than the covariance, as traditionally done in GBLUP for modeling the dominance effects. The use of Deep Kernels is also favored because the environmental kinship accounted for based on environmental distances due to nongenomic covariables, is regulated by the phenotypic information in the training set, thereby resulting in more accurate modeling of the envirotype-to-phenotype (E-to-P) dynamics in the prediction of new genotypes and new environments. This stems from the fact that indirectly, in the phenotype provided in the training population set, there is a genomic similarity relationship that determines the E-to-P relationship, part of which is captured by the genomic kernels and the rest by the environmental kernel. Despite these advantages, both the Gaussian Kernel and the Deep Kernel are faster, more accurate, and have a better resolution in predicting specific genotypes than the GBLUP models. In contrast with other reaction-norm proposals, such as the use of factorial regression to dissect E–P in secondary traits, the use of crop-growth models, and the use of envirotyping data to group environments and target WGP models, here we can use in a faster way the large-scale envirotypic data (*enviromics*) to explore alternative kinships across the benchmark genomic data.

### Large-scale genomics and enviromics with GK or DK

We demonstrate that the use of several sources of genomic variation (additive + dominance + GE interaction) guided by envirotyping is useful for increasing model accuracy. The use of the Gaussian kernel or Deep kernel makes it possible to capitalize on these effects, translating them into a drastic increase in PA, reduction of computational processing time, a greater explanation of phenotypic variation, and reduction of residual variation. New sources of nongenomic variation can be incorporated into WGP models through GK or DK to seek greater gains in PA under WGP–MET, as they are efficient in dealing with large-scale data. Here we also show that the use of environmental information through distribution quantiles is efficient for characterizing environments and, consequently, gives the kernels the ability to reproduce environmental similarities that can be explored in prediction. The field of large-scale enviromics still has a long pathway, but strategies that integrate E-to-P modeling are a bottleneck to overcome in genomic prediction, which benchmark GBLUP models are unable to achieve.

### Data repository

All data (phenotypic, genotypic, and envirotypic), Supplementary Material, and codes used in this study are available at https://github.com/gcostaneto/KernelMethods [verified 27 July, 2020].

On the previous link, there is a simplified tutorial of how the kernel methods and statistical models were programmed in R. We also connected the repositories of CIMMYT Dataverse [https://data.cimmyt.org/dataset.xhtml?persistentId=hdl:11529/10887, verified 20 May, 2020] and Mendeley [https://data.mendeley.com/datasets/tpcw383fkm/3, verified 20 May, 2020] where the full data sets of HEL and USP are available, respectively.

## Supplementary information

Supplementary material

## Data Availability

All analyses were conducted using R statistical software (R Core Team 2019). Data and codes are available at https://github.com/gcostaneto/KernelMethods [verified 27 July 2020].
