# Supplementary material for: Nonlinear kernels, dominance, and envirotyping data increase the accuracy of genome-based prediction in multi-environment trials
Source: Heredity (Edinb). 2020 Aug 27;126(1):92–106. doi: 10.1038/s41437-020-00353-1 (PMC7852533; doi:10.1038/s41437-020-00353-1)
Supplement: Supplementary file 1 — Supplementary material [file 41437_2020_353_MOESM1_ESM.docx]

**Deep kernels, dominance, and envirotyping data may boost the genome-based prediction in multi-environment trials**

Supplementary Figures and Tables

**Supplementary Table S1**. Geographic coordinates of each experimental site and environments used in both HEL and USP sets

| **Set** | **ID** | **Site** | **Region** | **Year** | **Management** | **Latitude** | **Longitude** |
| --- | --- | --- | --- | --- | --- | --- | --- |
| HEL | S1 | Nova Mutum | Middle-Western | 2015 | Potential Yield | -13.050 | -56.050 |
|  | S2 | Sorriso |  |  |  | -12.320 | -55.420 |
|  | S3 | Patos de Minas | Southwestern |  |  | -18.340 | -46.310 |
|  | S4 | Ipiaçú |  |  |  | -18.900 | -49.560 |
|  | S5 | Sertanópolis | South |  |  | -23.030 | -51.020 |
| USP | E1 | Piracicaba | Southwestern | 2016 | Low N Level | -22.705 | -47.637 |
|  | E2 |  |  |  | Ideal N Level | -22.705 | -47.637 |
|  | E3 | Anhumas |  |  | Low N Level | -22.870 | -47.997 |
|  | E4 |  |  |  | Ideal N Level | -22.870 | -47.997 |
|  | E5 | Piracicaba |  | 2017 | Low N Level | -22.705 | -47.637 |
|  | E6 |  |  |  | Ideal N Level | -22.705 | -47.637 |
|  | E7 | Anhumas |  |  | Low N Level | -22.870 | -47.997 |
|  | E8 |  |  |  | Ideal N Level | -22.870 | -47.997 |

**Supplementary Table S2**. Summary of the variance components (± standard deviations) for each model-kernel method combination in HEL maize set estimated from a Markov Chain involving 10,000 iterations. Bold numbers refer to the best models in reducing residual variation and exploring envirotype and dominance effects.

| **Kernel** | **Model** | **Source of Variation** | | | | | | | |
| --- | --- | --- | --- | --- | --- | --- | --- | --- | --- |
|  |  | **A** | **D** | **AE** | **DE** | **W** | **AW** | **DW** | **Residual** |
| **GB** | EA | 0.241 ± 0.046 |  |  |  |  |  |  | 0.952 ± 0.039 |
|  | EAD | 0.231 ± 0.044 | 0.148 ± 0.021 |  |  |  |  |  | 0.928 ± 0.039 |
|  | EAD+GE | **0.234 ± 0.045** | **0.143 ± 0.021** | **0.147 ± 0.02** | **0.307 ± 0.053** |  |  |  | **0.650 ± 0.053** |
|  | EADW | 0.211 ± 0.038 | 0.130 ± 0.016 |  |  | 0.501 ± 0.240 |  |  | 0.232 ± 0.016 |
|  | EADW+GW | **0.210 ± 0.040-** | **0.129 ± 0.016** |  |  | **0.474 ± 0.222** | **0.023 ± 0.004** | **0.079 ± 0.01** | **0.186 ± 0.016** |
| **GK** | EA | 0.274 ± 0.044 |  |  |  |  |  |  | 0.928 ± 0.039 |
|  | EAD | 0.231 ± 0.040 | 0.181 ± 0.028 |  |  |  |  |  | 0.929 ± 0.038 |
|  | EAD+GE | **0.210 ± 0.035** | **0.159 ± 0.022** | **0.235 ± 0.022** | **0.121 ± 0.013** |  |  |  | **0.056 ± 0.007** |
|  | EADW | 0.216 ± 0.035 | 0.162 ± 0.022 |  |  | 0.586 ± 0.526 |  |  | 0.256 ± 0.011 |
|  | EADW+GW | **0.220 ± 0.038** | **0.163 ± 0.022** |  |  | **0.441 ± 0.236** | **0.012 ± 0.003** | **0.026 ± 0.012** | **0.244 ± 0.012** |
| **DK** | EA | 0.621 ± 0.128 |  |  |  |  |  |  | 0.922 ± 0.039 |
|  | EAD | 0.464 ± 0.104 | 0.094 ± 0.016 |  |  |  |  |  | 0.927 ± 0.039 |
|  | EAD+GE | **0.401 ± 0.082** | **0.087 ± 0.013** | **0.909 ± 0.088** | **0.062 ± 0.007** |  |  |  | **0.053 ± 0.007** |
|  | EADW | 0.406 ± 0.093 | 0.087 ± 0.012 |  |  | 1.105 ± 0.602 |  |  | 0.257 ± 0.011 |
|  | EADW+GW | **0.458 ± 0.095** | **0.089 ± 0.013** |  |  | **1.527 ± 1.822** | **0.007 ± 0.006** | **0.003 ± 0.001** | **0.256 ± 0.011** |

**Supplementary Table S3**. Summary of the variance components (± standard deviations) for each model-kernel method combination in USP maize set estimated from a Markov Chain involving 10,000 iterations. Bold numbers refer to the best models in reducing residual variation and exploring envirotype and dominance effects

| **Kernel** | **Model** | **Source of Variation** | | | | | | | |
| --- | --- | --- | --- | --- | --- | --- | --- | --- | --- |
|  |  | **A** | **D** | **AE** | **DE** | **W** | **AW** | **DW** | **Residual** |
| **GB** | EA | 1.164 ± 0.253 |  |  |  |  |  |  | 3.326 ± 0.071 |
|  | EAD | 1.257 ± 0.265 | 0.422 ± 0.044 |  |  |  |  |  | 3.051 ± 0.068 |
|  | EAD+GE | 1.331 ± 0.294 | 0.421 ± 0.045 | 0.339 ± 0.036 | 0.358 ± 0.039 |  |  |  | 2.712 ± 0.070 |
|  | EADW | **1.327 ± 0.319** | **0.516 ± 0.047** |  |  | 2.154 ± 1.687 |  |  | 1.522 ± 0.092 |
|  | EADW+GW | **1.338 ± 0.288** | **0.557 ± 0.049** |  |  | 2.729 ± 2.261 | 0.058 ± 0.009 | **0.220 ± 0.021** | **1.181** ± 0.117 |
| **GK** | EA | **1.631 ± 0.215** |  |  |  |  |  |  | 3.090 ± 0.068 |
|  | EAD | 1.063 ± 0.155 | 0.521 ± 0.064 |  |  |  |  |  | 3.046 ± 0.066 |
|  | EAD+GE | **1.247 ± 0.192** | **0.676 ± 0.076** | **0.586 ± 0.055** | **0.489 ± 0.051** |  |  |  | **1.092** ± 0.039 |
|  | EADW | 1.189 ± 0.171 | 0.626 ± 0.072 |  |  | 7.827 ± 9.810 |  |  | 1.601 ± 0.036 |
|  | EADW+GW | 1.143 ± 0.206 | 0.623 ± 0.074 |  |  | 3.213 ± 4.654 | 0.148 ± 0.027 | 0.146 ± 0.052 | 1.394 ± 0.035 |
| **DK** | EA | 1.191 ± 0.169 |  |  |  |  |  |  | 3.100 ± 0.069 |
|  | EAD | 0.713 ± 0.114 | 0.435 ± 0.057 |  |  |  |  |  | 3.046 ± 0.067 |
|  | EAD+GE | **0.816 ± 0.128** | **0.602 ± 0.073** | **0.377 ± 0.041** | **0.418 ± 0.049** |  |  |  | **1.121** ± 0.042 |
|  | EADW | **0.838 ± 0.143** | **0.548 ± 0.065** |  |  | 3.177 ± 1.875 |  |  | 1.602 ± 0.035 |
|  | EADW+GW | 0.762 ± 0.151 | 0.549 ± 0.072 |  |  | 0.690 ± 0.603 | 0.093 ± 0.015 | 0.015 ± 0.006 | 1.441 ± 0.034 |

**
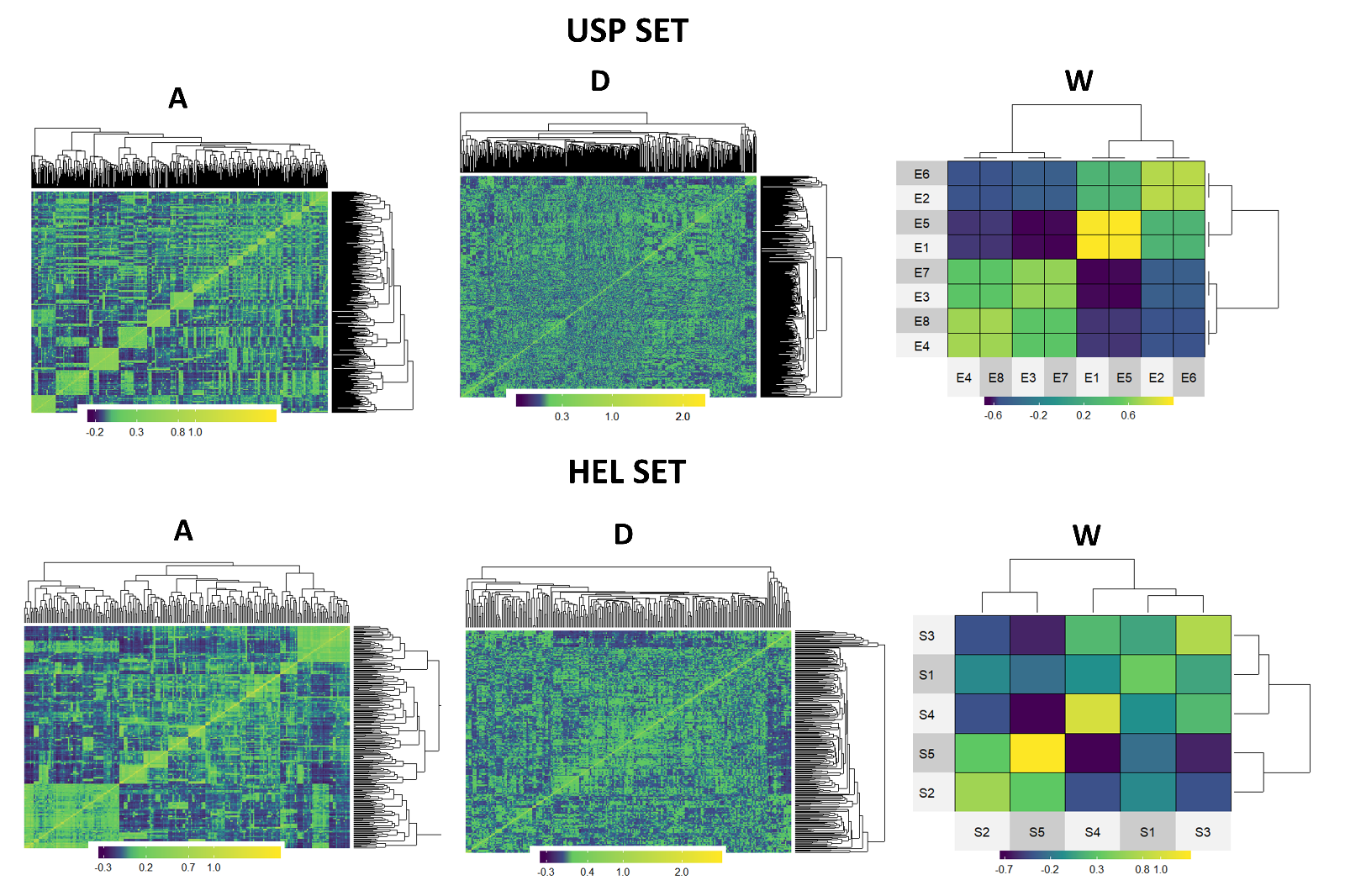
**

**Supplementary Figure S1** Relationship Kernels (variance-covariance matrix) for additive (A), dominance (D) and envirotyping data (W) based on GBLUP/GB kernel method for two maize sets (USP and HEL).

**
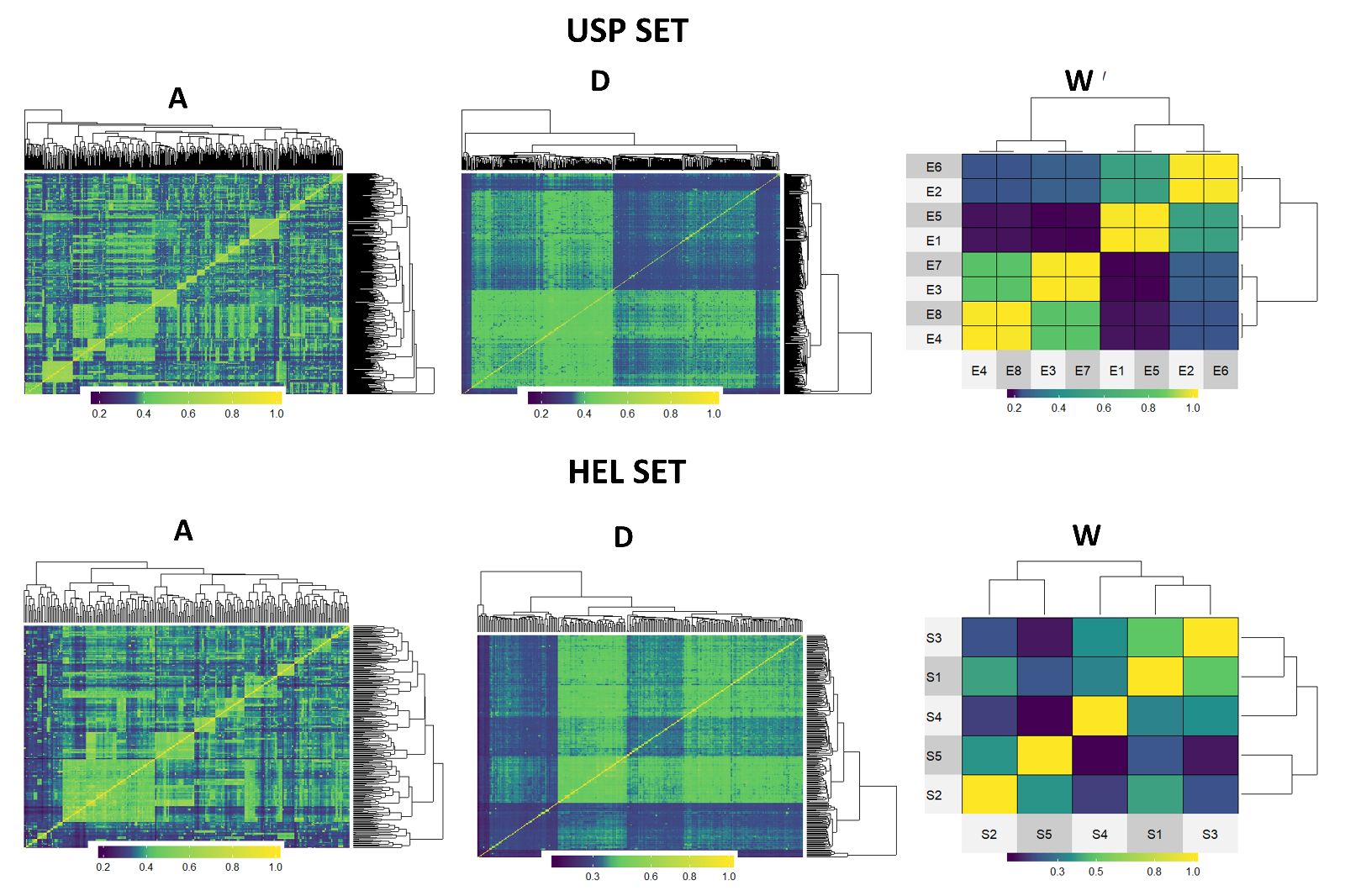
**

**Supplementary Figure S2** Relationship Kernels (variance-covariance matrix) for additive (A), dominance (D) and envirotyping data (W) based on Gaussian kernel (GK) method f or two maize sets (USP and HEL).

**
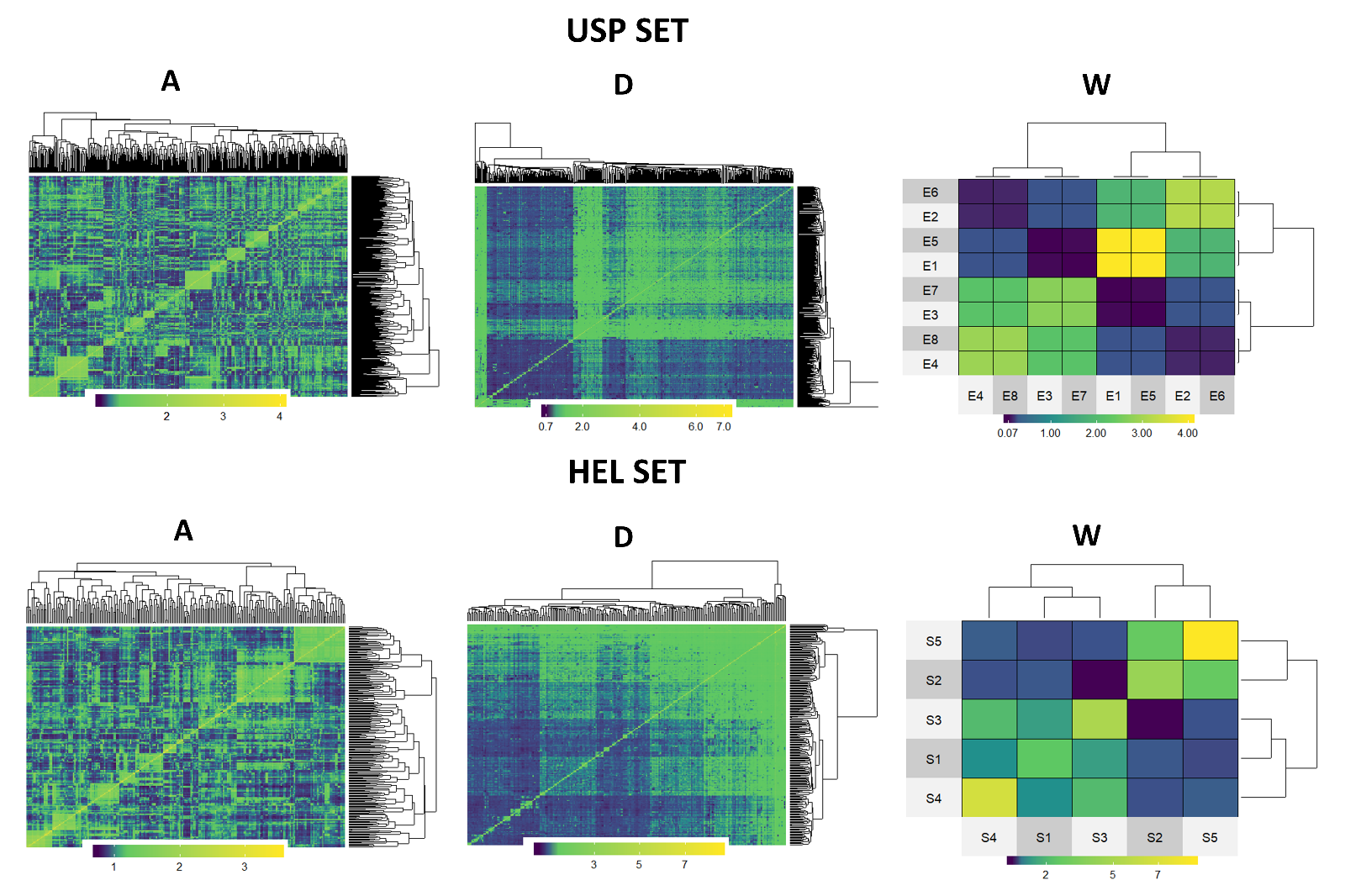
**

**Supplementary Figure S3** Relationship Kernels (variance-covariance matrix) for additive (A), dominance (D) and envirotyping data (W) based on Deep kernel (DK) method for two maize sets (USP and HEL).
